# Sleep patterns modify the association of 25(OH)D with poor cardiovascular health in pregnant women

**DOI:** 10.3389/fnut.2022.1013960

**Published:** 2022-11-14

**Authors:** Wan-jun Yin, Li-jun Yu, Peng Wang, Rui-xue Tao, Xiao-min Jiang, Ying Zhang, Dao-min Zhu, Peng Zhu

**Affiliations:** ^1^Department of Maternal, Child and Adolescent Health, School of Public Health, Anhui Medical University, Hefei, China; ^2^MOE Key Laboratory of Population Health Across Life Cycle, Hefei, China; ^3^NHC Key Laboratory of Study on Abnormal Gametes and Reproductive Tract, Hefei, China; ^4^Anhui Provincial Key Laboratory of Population Health and Aristogenics, Anhui Medical University, Hefei, China; ^5^Department of Obstetrics and Gynecology, Hefei First People’s Hospital, Hefei, China; ^6^Department of Obstetrics and Gynecology, Anhui Province Maternity and Child Health Hospital, Hefei, China; ^7^Department of Obstetrics and Gynecology, The First Affiliated Hospital of Anhui Medical University, Hefei, China; ^8^Department of Sleep Disorders, Affiliated Psychological Hospital of Anhui Medical University, Hefei, China; ^9^Hefei Fourth People’s Hospital, Hefei, China; ^10^Anhui Mental Health Center, Hefei, China

**Keywords:** vitamin D, sleep patterns, cardiovascular health, pregnant women, birth cohort

## Abstract

**Background:**

The relationship between vitamin D status and gestational cardiovascular health (CVH) is inconsistent in previous studies. Emerging evidence shows that sleep behaviors are related to vitamin D metabolism. However, no studies evaluate the interaction of vitamin D and sleep behaviors on gestational CVH.

**Objective:**

We aimed to estimate the relationship between 25-hydroxyvitamin D [25(OH)D] concentrations and gestational CVH, and whether the relationship was modified by sleep behaviors.

**Methods:**

The data of this study was from a multicenter birth cohort study. A total of 9,209 pregnant women at 16–23 weeks of gestation were included. 25(OH)D concentrations were measured from collected blood. Sleep patterns consisted of major sleep behaviors including duration, chronotype, insomnia, snoring, and excessive daytime sleepiness. Data on poor CVH was based on four “clinical” CVH metrics, including body mass index, blood pressure, total cholesterol, and glucose levels.

**Results:**

The proportion of women with poor CVH was 25.0%. The relative risk (RR) (95%CI) of poor CVH was 0.67 (0.58–0.76) in women with 25(OH)D ≥ 50 nmol/L after multivariate adjustments. Lower 25(OH)D concentrations were significantly associated with poor CVH. Such association was also evident in subgroups analysis. We found a significant interaction of 25(OH)D (*P* for interaction = 0.01) with sleep patterns on the risk of poor CVH. A negative dose-response relation was observed between 25(OH)D concentrations and poor CVH risk in healthy or intermediate sleep, not poor sleep. 25(OH)D concentrations were lower and the risk of poor CVH was higher in pregnant women with poor sleep patterns (*P* < 0.05).

**Conclusion:**

Our study suggests that sleep patterns modify the association of 25(OH)D concentrations with the CVH among pregnant women.

## Introduction

Pregnancy is increasingly recognized as a critical period to address female lifetime cardiovascular health (CVH) (1). Exposure to cardiovascular risk factors (obesity, hyperglycemia, hypertension, hyperlipidemia) during pregnancy may lead to a higher risk trajectory for cardiovascular disease (CVD) in females (2–4). Thus, primary preventive CVD interventions applicable to broad pregnant women are needed, and increasing 25-hydroxyvitamin D [25(OH)D] concentrations may be an attractive strategy.

Within the last 1 or 2 decades, there is growing recognition that vitamin D plays an important role in CVD prevention and treatment. Several studies have explored the association of vitamin D with CVD (5–9). However, the impacts of vitamin D supplementation on CVH were mixed across interventions (5, 6, 10). In addition, a meta-analysis (11) of prospective studies amassed that an increased risk of CVD is associated with low serum 25(OH)D concentrations, yet significant heterogeneity was detected in these studies. Inconsistent results could partly be attributed to lifestyle variations, such as sleep behaviors, related to CVD and vitamin D status across the evidence.

Significant evidence has indicated the association between various sleep behaviors and vitamin D metabolism (12, 13). Several observational meta-analyses (14, 15) showed obstructive sleep apnea (OSA) and sleep disorders were associated with lower 25(OH)D concentrations. There is evidence in showing there are vitamin D receptors in the brain involved in the sleep-wake cycle (16), providing a mechanistic explanation for how vitamin D deficiency can contribute to sleep disturbances. Notably, emerging evidence suggests that poor sleep behaviors were also associated with an increased risk of CVD (17–19). Therefore, we hypothesized that sleep patterns modify the association of 25(OH)D concentrations with the CVH among pregnant women.

We conducted a prospective birth cohort study to investigate the association between serum 25(OH)D and CVH in pregnant women and particularly assessed the potential modifying effect of a combination of major sleep behaviors (duration, chronotype, insomnia, snoring, and excessive daytime sleepiness), which was characterized by a healthy sleep score. We also aimed to estimate the modifying effect of the healthy sleep score on vitamin D supplementation behavior and CVH of these women.

## Materials and methods

### Study participants and design

This study was conducted based on the Maternal and Infant Health cohort study in Hefei (MIH-Hefei) (20) comprising 9,886 pregnant women in Hefei (32°N latitude), China. Participants aged 18–45 years were recruited from March 2015 to October 2021 across Anhui Women and Child Health Care Hospital, the First People’s Hospital of Hefei City, and the First Affiliated Hospital of Anhui Medical University. The inclusion criteria of this study included 16–23 weeks of gestation, no communication problems, no assisted conception, delivered at the recruited hospital, and singleton pregnancy.

At recruitment, A face-to-face interview or medical records were used to collect data on sociodemographic characteristics, perinatal health status, lifestyle, dietary habits, vitamin D supplementation status, and sleep behaviors. After that, trained staff collected venous blood.

The exclusion criteria were the following: serious pregnancy complications (e.g., heart failure, severe anemia); abnormal liver, renal, or thyroid function; missing blood samples, and unavailable gestational CVH data. Given that blood pressure (BP) is a component of CVH, women with eclampsia or preeclampsia were not excluded. Finally, full data were obtained for 9 209 pregnant women (Supplementary Figure 1). The study was approved by the Ethics Committee of Anhui Medical University (NO. 2015002). The study was conducted according to all relevant guidelines and regulations, and each participant provided written informed consent.

### Measurement of serum 25(OH)D

Venous blood was collected at 16–23 weeks of gestation. The centrifuged serum sample was promptly refrigerated at 4°C, then placed in an −80°C freezer within 8 h. Single measurements were performed for serum 25(OH)D by well-trained staff at the Ministry of Education Key Laboratory of Population Health Across Life Cycle using a commercial chemiluminescence immunoassay (DiaSorin Stillwater, MN, USA). The intra-assay and inter-assay coefficients of variation were 8.8 and 11.1%, respectively. In addition, 25(OH)D concentrations were divided into five groups by the quintile (Q1 < *P*_20_, *P*_20_ ≤ Q2 < *P*_40_, *P*_40_ ≤ Q3 < *P*_60_, *P*_60_ ≤ Q4 < *P*_80_, Q5 ≥ *P*_80_).

### Assessment of sleep patterns

At recruitment (16–23 weeks of gestation), sleep habits of 9,209 pregnant women over the past month were collected with a single shot by a standard questionnaire. The sleep patterns were derived from self-reported records and included five sleep behaviors (duration, chronotype, insomnia, snoring, and excessive daytime sleepiness)(17). The following are the definitions of healthy sleep behaviors during pregnancy: 7–8 h of sleep per day, no excessive daytime sleepiness (the score of Epworth Sleeping Scale ≤ 10 points) (21), never or rarely insomnia, no snoring, and early chronotype (morning or morning then evening). For each sleep factor, healthy behaviors were coded as 1, and the others were coded as 0. The healthy sleep score was composed of those five sleep factors varying from 0 to 5. The overall sleep patterns were defined as “poor sleep” (scores < 3), “intermediate sleep” (scores of 3 or 4), and “healthy sleep” (scores of 5).

### Assessment of gestational cardiovascular health

Four “clinical” CVH metrics were used to assess gestational CVH [body mass index (BMI), BP, total cholesterol (TC) level, and, glucose level] (22). Each CVH metric was rated as ideal (2 points), intermediate (1 point), or poor (0 points). The following are the detailed classification criteria. Ideal BMI: < 28.4 kg/m^2^, intermediate BMI: 28.5–32.9 kg/m^2^, poor BMI: ≥ 33 kg/m^2^. Ideal BP: systolic blood pressure (SBP) < 120 mm Hg and diastolic blood pressure (DBP) < 80 mm Hg; intermediate BP: SBP 120–139 mm Hg or DBP 80–89 mm Hg; poor BP: SBP ≥ 140 mm Hg or DBP ≥ 90 mm Hg. Ideal TC: < 260 mg/dL, intermediate TG: 260–299 mg/dL, and poor TG: ≥ 300 mg/dL. glucose: ideal: non-gestational diabetes mellitus (GDM), poor: diagnosis of GDM. (22) At 24–28 gestational weeks, we obtained the results of four “clinical” CVH metrics in the hospitals (BMI, BP, TC, and blood glucose). Poor CVH was defined as having one or more of these indicators as poor.

### Potential confounders

In this study, the potential confounder was age (<30 and ≥ 30 years), prepregnancy BMI (< 24 and ≥ 24 kg/m^2^), gestational weight gain (GWG) rate (< *P*_75_ and ≥ *P*_75_, kg/w), family history of diabetes, hypertension, CVD (including myocardial infarction, coronary heart disease, stroke), daily outdoor time, sun-bathing, vitamin D supplementation frequency, the season of blood collection, and dietary vitamin D intake habits (sea-fish, egg, milk, fungi, red meat, and white meat intake) (23, 24) which have previously been linked to CVH or 25(OH)D concentrations in pregnant women. The exact dose of vitamin D supplementation was not available. According to reports of pregnant women on vitamin D supplementation, the dose ranged from 400 IU to 600 IU daily. The dietary vitamin D intake habits was from food frequency questionnaires. GWG rate was the following: (current weight- self-reported prepregnancy weight)/current gestational week. Depression in pregnant women was screened using the Edinburgh Postpartum Depression Scale (points ≥ 10). Other potential confounders included education (12 and > 12 years of completed schooling), household income (less than 6,000 RMB or more than 6,000 RMB), parity (primipara or multipara), residence (urban or non-urban), physical activity, and husband smoking during pregnancy. Often activity was defined as physical activity (including table tennis, badminton, and vigorous walking) for at least 30 min per day.

### Statistical analysis

A *t*-test was used to compare the difference in 25(OH)D concentrations among all groups of characteristics. The relationship between 25(OH)D concentrations and poor CVH across the different healthy sleep scores was fitted in logistics regression models. Models were adjusted for age, education, household income, residence, the season of blood collection, prepregnancy BMI, GWG rate, parity, depression, family history of diabetes, hypertension, and CVD, smoking, husband’s smoking, daily outdoor time, sun exposure, activities, dietary vitamin d intake habits (sea-fish, egg, milk, fungi, red meat, and white meat intake), vitamin D supplementation.

Stratified analyses were used to estimate the association of 25(OH)D concentrations in quintiles with poor CVH or each individual “clinic index” according to healthy sleep patterns. The likelihood ratio test comparing models was used to estimate the interaction test between 25(OH)D concentrations and sleep patterns. All statistical analyses were conducted in SPSS 23.0 (IBM Corp., Armonk, NY, USA).

## Results

The distributions of sociodemographic characteristics, perinatal health status, lifestyle, and dietary vitamin D intake habits of participants did not differ from those who withdrew, according to attrition analyses. The average participant age was 28.5 years [standard deviation (SD) = 3.8], the mean prepregnancy BMI was 21.3 [SD = 2.9]) kg/m^2^, and the mean concentrations of 25(OH)D were 40 [SD = 18]) nmol/L. The proportion of women with poor CVH was 25.0%.

Table 1 shows the characteristics of the study population across the differences in 25(OH)D concentrations. Participants with higher 25(OH)D were higher educational attainment, blood collection in summer or fall, lower prepregnancy BMI, lower GWG rate, more outdoor time, frequent sunbathing, frequent vitamin D supplementation, and more frequent egg, milk, or white meat consumption.

**TABLE 1:** Characteristics of the study population.

Variables	*N* (%)	25(OH)D, nmol/L *M* ± SD	*P*-value^a^
**Sociodemographic characteristics**			
Age < 30 years	5,408 (58.7)	40 ± 18	0.01
Age ≥ 30 years	3,801 (41.3)	40 ± 19	
Education ≤ 12 years	3,529 (38.3)	39 ± 18	0.01
Education > 12 years	5,680 (61.7)	40 ± 19	
Urban residence	8,342 (90.6)	39 ± 18	0.68
Non-urban residence	867 (9.4)	40 ± 19	
Household income < 6,000 yuan	2,712 (29.4)	40 ± 18	0.30
Household income ≥ 6,000 yuan	6,497 (70.6)	40 ± 19	
Winter or spring	4,542 (49.3)	37 ± 18	<0.001
Summer or fall	4,667 (50.7)	43 ± 19	
**Perinatal health status**			
Prepregnancy BMI < 24 kg/m^2^	7,574 (82.2)	41 ± 19	<0.001
Prepregnancy BMI ≥ 24 kg/m^2^	1,635 (17.8)	37 ± 17	
GWG rate < *P*_75_, kg/w	6,864 (74.5)	40 ± 19	<0.001
GWG rate ≥ *P*_75_, kg/w	2,345 (25.5)	39 ± 18	
Multipara	4,320 (46.9)	40 ± 18	0.16
Primipara	4,889 (53.1)	40 ± 19	
Depression	3,095 (33.6)	40 ± 18	0.18
Non-depression	6,114 (66.4)	40 ± 18	
Non-family history of diabetes	8,352 (90.7)	40 ± 18	0.48
Family history of diabetes^b^	857 (9.3)	40 ± 18	
Non-family history of hypertension	6,098 (66.2)	40 ± 18	0.20
Family history of hypertension ^b^	3,111 (33.8)	40 ± 19	
Non-family history of CVD	8818 (95.8)	40 ± 18	0.44
Family history of CVD^b^	391 (4.2)	41 ± 20	
**Pregnancy lifestyle factors**			
Husband smoking	3,819 (41.5)	40 ± 18	0.14
Husband no smoking	5,390 (58.5)	40 ± 19	
Outdoor time^c^ < 1 h/d	6,206 (67.4)	39 ± 18	<0.001
Outdoor time ≥ 1 h/d	3,003 (32.6)	41 ± 18	
Often sun exposure	1,120 (12.2)	43 ± 19	<0.001
Rare sun exposure	8,089 (87.8)	40 ± 18	
Often activities	4,088 (44.4)	40 ± 18	0.25
Rare activities	5,121 (55.6)	40 ± 18	
Vitamin D supplementation < 3 times/w	4,053 (44.0)	35 ± 14	<0.001
Vitamin D supplementation ≥ 3 times/w	5,156 (56.0)	43 ± 20	
**Dietary vitamin D intake habits**			
Sea-fish intake < 3 times/w	9,046 (98.2)	40 ± 18	0.56
Sea-fish intake ≥ 3 times/w	163 (1.8)	41 ± 18	
Egg intake < 3 times/w	2,350 (25.5)	38 ± 17	<0.001
Egg intake ≥ 3 times/w	6,859 (74.5)	41 ± 19	
Milk intake < 3 times/w	3,816 (41.4)	39 ± 18	<0.001
Milk intake ≥ 3 times/w	5,393 (58.6)	41 ± 19	
Fungi intake < 3 times/w	8,746 (95.0)	40 ± 18	0.80
Fungi intake ≥ 3 times/w	463 (5.0)	40 ± 19	
Red meat intake < 3 times/w	2169 (23.6)	40 ± 18	0.34
Red meat intake ≥ 3 times/w	7,039 (76.4)	40 ± 18	
White meat intake < 3 times/w	7,063 (76.7)	40 ± 18	0.01
White meat intake ≥ 3 times/w	2,145 (23.3)	41 ± 19	

CVD, cardiovascular disease; GWG, gestational weight gain.

^a^Based on the *t*-test; ^b^Family history of diabetes, hypertension, or CVD was defined as either parent having diabetes, hypertension, or CVD; ^c^Outdoor time means time spent outdoors in the daytime.

Lower 25(OH)D concentrations were found to be significantly associated with poor CVH risk in the multivariate-adjusted models (Table 2). A negative dose-response relationship was observed between 25(OH)D concentrations and poor CVH risk. Such association was also evident in subgroups subgroup analysis. The RR (95% CI) of poor CVH was 0.67 (0.58–0.76) in women with 25(OH)D ≥ 50 nmol/L, and the highest quintile of 25(OH)D had a 55% lower poor CVH risk than the lowest quintile.

**TABLE 2:** Adjusted RRs and 95%CI for serum 25(OH)D with poor CVH, overall and by subgroup.

	Serum 25(OH)D, RR (95% CI)						
	
Groups	Q1 (<25 nmol/L)	Q2 (25∼32 nmol/L)	Q3 (33∼41 nmol/L)	Q4 (42∼55 nmol/L)	Q5 (≥56 nmol/L)	RR (95% CI) for ≥ 50 nmol/L vs. < 50 nmol	*P* for interaction
Case subjects/*N*	588/1,839	504/1,842	474/1,844	432/1,843	301/1,841	–	
Overall^a^	1.00	0.81 (0.70, 0.94)	0.75 (0.64, 0.87)	0.64 (0.55, 0.75)	0.45 (0.38, 0.54)	0.67 (0.58, 0.76)	
Age^b^							0.97
<30 years	1.00	0.81 (0.66, 0.99)	0.75 (0.62, 0.92)	0.66 (0.53, 0.81)	0.43 (0.34, 0.55)	0.63 (0.52, 0.76)	
≥30 years	1.00	0.83 (0.66, 1.03)	0.75 (0.60, 0.94)	0.63 (0.50, 0.80)	0.47 (0.36, 0.61)	0.71 (0.58, 0.86)	
Parity^b^							
Multipara	1.00	0.87 (0.71, 1.08)	0.83 (0.67, 1.03)	0.74 (0.59, 0.93)	0.57 (0.46, 0.73)	0.72 (0.60, 0.87)	0.10
Primipara	1.00	0.77 (0.62, 0.94)	0.68 (0.55, 0.84)	0.57 (0.46, 0.71)	0.35 (0.28, 0.46)	0.62 (0.51, 0.75)	
Depressive position^b^							0.28
Depression	1.00	0.74 (0.62, 0.89)	0.70 (0.59, 0.84)	0.65 (0.54, 0.79)	0.43 (0.35, 0.54)	0.71 (0.61, 0.84)	
Non-depression	1.00	0.97 (0.75, 1.26)	0.85 (0.66, 1.12)	0.63 (0.48, 0.83)	0.49 (0.36, 0.66)	0.58 (0.46, 0.74)	
Season^b^							0.79
Winter or spring	1.00	0.83 (0.69, 1.02)	0.80 (0.65, 0.98)	0.63 (0.50, 0.79)	0.41 (0.31. 0.54)	0.58 (0.47, 0.72)	
Summer or fall	1.00	0.78 (0.62, 0.98)	0.70 (0.56, 0.88)	0.66 (0.53, 0.82)	0.48 (0.37, 0.61)	0.75 (0.63, 0.90)	

CVH, cardiovascular health; CI, confidence interval; Q, quintile.

^a^Adjusted for age, education, household income, residence, the season of blood collection, prepregnancy BMI, gestational weight gain rate, parity, depression, family history of diabetes, hypertension, and CVD, smoking, husband’s smoking, daily outdoor time, sun exposure, activities, dietary vitamin d intake habits (sea-fish, egg, milk, fungi, red meat, and white meat intake), vitamin D supplementation.

^b^Adjusted for above factors, except stratification factor.

**FIGURE 1:**
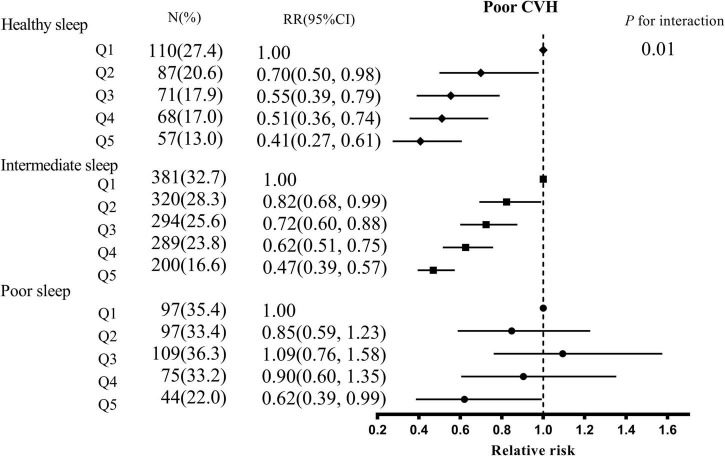
The association between serum 25(OH)D concentrations and poor CVH stratified by overall sleep patterns. CVH, cardiovascular health; CI, confidence interval; Q, quintile. Adjusted for age, education, household income, residence, the season of blood collection, prepregnancy BMI, GWG rate, parity, depression, family history of diabetes, hypertension, and CVD, smoking, husband’s smoking, daily outdoor time, sun exposure, activities, dietary vitamin d intake habits (sea-fish, egg, milk, fungi, red meat, and white meat intake), vitamin D supplementation.

The stratified analysis showed whether sleep patterns modified the association between 25(OH)D and the risk of poor CVH according to the healthy sleep scores (Figure 1). The interaction between 25(OH)D and sleep patterns on the risk of poor CVH was significant (*P* for interaction = 0.01). In healthy or intermediate sleep, 25(OH)D was inversely related to the risk of poor CVH in a dose-response fashion. There was no significant negative correlation of poor CVH for quintile 2–4 groups compared with quintile 1 group of 25(OH)D among poor sleep. The association between 25(OH)D and GDM, high BP, high total cholesterol, or overweight was similar to that of poor CVH (Supplementary Figure 2).

Table 3 shows the association of sleep patterns with serum 25(OH)D concentrations and poor CVH. We found that 25(OH)D concentrations were lower and the risk of poor CVH was higher in pregnant women with poor sleep patterns (*P* < 0.05). We also found similar results in the association of sleep behaviors (sleep duration, daytime sleepiness, insomnia, snoring) with serum 25(OH)D concentrations and poor CVH (Supplementary Table 1).

**TABLE 3:** Association of sleep patterns with serum 25(OH)D concentrations and poor CVH.

Sleep patterns	25(OH)D		Poor CVH		
		
	M ± SD, nmol/L	*P*-value^a^	*N* (%)	*P*-value^b^	RR (95%CI)^c^
Healthy sleep	41 ± 18	<0.001	393 (19.1)	< 0.001	1.00
Intermediate sleep	40 ± 19		1,484 (25.3)		1.46 (1.29, 1.67)
Poor sleep	38 ± 18		442 (32.7)		2.04 (1.72, 2.41)

CVH, cardiovascular health; CI, confidence interval.

^a^Based on one-way ANOVA; ^b^Based on chi-square test. ^c^Based on logistics regression models and adjusted for age, education, household income, residence, the season of blood collection, prepregnancy BMI, gestational weight gain rate, parity, depression, family history of diabetes, hypertension, and CVD, smoking, husband’s smoking, daily outdoor time, sun exposure, activities, dietary vitamin d intake habits (sea-fish, egg, milk, fungi, red meat, and white meat intake), vitamin D supplementation.

## Discussion

To our knowledge, this is the first study to assess the effect of sleep behaviors on the association between 25(OH)D and gestational CVH. A negative dose-response association was observed between 25(OH)D concentrations and poor CVH risk, independent of traditional risk factors. In addition, we also discovered that sleep patterns might significantly modify such association, while the decreased poor CVH risk associated with high serum 25(OH)D concentrations appeared to be attenuated among participants with poor sleep patterns.

Consistent with our findings, several observational studies (8, 9, 25) showed that serum 25(OH)D were inversely associated with poor CVH risk, and previous randomized controlled trials (RCTs) also observed that vitamin D supplementation improved some CVH markers. (10, 26) Although the mechanisms underlying the observed inverse relationship between serum 25(OH)D and CVD risk remain unknown, several potential explanations exist. For example, vitamin D deficiency might activate the renin-angiotensin-aldosterone system and increase the CVD risk (27). A recent RCT also suggested that supplementation with high-dose vitamin D3 in patients with grades I-II essential hypertension reduces BP (28). In addition, a previous animal study showed that adequate 25(OH)D may decrease CVD risk through anti-inflammatory processes (29). 25(OH)D may control immunological and inflammatory cell proliferation, differentiation, and function, increasing anti-inflammatory pathways and decreasing the activation of these cells (30).

For the first time, we observed that sleep patterns could modify the association of 25(OH)D with gestational CVH. We also discovered that healthy sleep patterns, such as sleep 7–8 h per day, no excessive daytime sleepiness, never or rarely insomnia, no snoring, and early chronotype were associated with a decreased risk of poor gestational CVH, including the risk of obesity, hyperglycemia, hypertension, hyperlipidemia. Notably, previous meta-analyses found significant heterogeneity in the associations of 25(OH)D with CVD across studies (11). RCTs (10, 31) also yielded inconclusive results regarding whether vitamin D supplementation may reduce the risk of CVD. Moreover, the evidence from the Mendelian randomization study (32) has shown that population-wide correction of vitamin D deficiency could reduce the burden of CVD. Our findings indicate that variable sleep behaviors may account for some of this heterogeneity, and healthy sleep patterns may reinforce the validity of the association of 25(OH)D with poor CVH in pregnant women.

Poor sleep patterns may modify the association between vitamin D and CVH through inflammatory pathways. Prior studies (33) have noted the association between poor sleep and increased inflammation, and chronic inflammation was a potential mechanism of CVD. The data (34) from the National Health and Nutrition Examination Survey have shown the reduction of inflammatory diet may attenuate the adverse effects of poor sleep on CVD. Our previous study (35) has found that adequate 25(OH)D plays an essential anti-inflammatory role in gestational CVH. So, poor sleep patterns may attenuate the positive effects of adequate 25(OH)D on CVH by increasing inflammation.

Our results of the interactions between 25(OH)D and sleep behaviors were biologically plausible. The main pathway generating 25(OH)D is endogenously synthesized via skin exposure to sunlight (36). Unhealthy sleep behaviors may have an impact on the amount of skin exposed to sunlight, leading to vitamin D deficiency (37). For example, daytime sleepiness may be associated with reduced outside activities and sun exposure, which reduced circulating 25(OH)D. Animal studies also showed that chronic sleep deficiency in rats significantly reduced circulating 25(OH)D concentrations (38). Furthermore, vitamin D deficiency may influence sleep patterns. Previous observational study found a relationship between 25(OH)D concentrations and daytime sleepiness. (39) An RCT (40) on US veterans with 25(OH)D concentrations < 75 nmol/L and short sleep duration (slightly over 4.5 h) showed approximately 0.75 h longer sleep duration after supplementation with vitamin D 1200 IU daily or 50 000 IU weekly for 3 months. Another RCT of vitamin D supplementation in patients with abnormal sleep suggests direct central effects of vitamin D on sleep (41). Moreover, the inverse associations between sleep behaviors including unhealthy sleep duration, or excessive daytime sleepiness, and 25(OH)D were also detected. An updated meta-analysis study (42) showed that OSA, as manifested by snoring symptoms, was a partial contributor to low serum 25(OH)D concentrations. The previous study (43) also suggested that patients with more severe OSA indices had lower 25(OH)D concentrations associated with abnormal glucose metabolism.

### Strengths and limitations

To our knowledge, this is the first study to evaluate weather sleep behaviors modify the association of 25(OH)D with gestational CVH. The large sample size and prospective design are two of this study’s major strengths. More importantly, a wide range of covariates was collected, including lifestyle, dietary habits, and sun exposure, allowing for rigorous confounding adjustment. However, a few potential restrictions need to be addressed. First, as we used self-reported sleep data in this investigation, exposures may have been incorrectly classified. Second, the healthy sleep score did not account for other sleep behavior such restless legs syndrome, which may interact with vitamin D on the gestational CVH. In addition, a note of caution should be exercised while interpreting our study. According to multiple literature data (44, 45), other components of the CVH score in this study (BP, BMI, TC, and glucose levels) are in inverse correlation with 25(OH)D concentrations. Therefore, higher CVH score will be automatically associated with higher 25(OH)D concentrations. Third, because this investigation was observational in nature, causality could not be established. Randomized clinical trials are required to validate our findings. Fourth, even though we rigorously eliminated a number of possible confounders from the analyses, such as lifestyle, sun exposure, and diet factors, residual confounding may still exist. Finally, because the current study was based on data from just one city, it is important to use caution when extrapolating the findings to other communities.

## Conclusion

In conclusion, our study observes that lower 25(OH)D concentrations are related to poor gestational CVH, and such associations are modified by sleep patterns. Our findings suggest that future RCTs of vitamin D supplementation need to consider the importance of lifestyle factors such as sleep behaviors in the prevention of cardiovascular diseases.

## Data availability statement

The raw data supporting the conclusions of this article will be made available by the authors, without undue reservation.

## Ethics statement

The studies involving human participants were reviewed and approved by the Ethics Committee of Anhui Medical University (No. 2015002). The patients/participants provided their written informed consent to participate in this study.

## Author contributions

W-JY performed the experiments and was responsible for the collection and compilation of data, analysis of data, and wrote the manuscript. PW contributed to the compilation of the data and helped write the manuscript. L-JY was responsible for the collection of clinical data and contributed to clinical assessments. X-MJ and YZ designed the study and assisted with data collection. PZ and D-MZ were the guarantor of this work designed and supervised the study, and revised the manuscript. All authors read and approved the final manuscript.

## Abbreviations

25(OH)D: 25-hydroxyvitamin D
BMI: body mass index
CVD: cardiovascular disease
CVH: cardiovascular health
DBP: diastolic blood pressure
GDM: gestational diabetes mellitus
GWG: gestational weight gain
OGTT: oral glucose tolerance test
OSA: obstructive sleep apnea
RCT: randomized controlled trial
SBP: systolic blood pressure
TC: total cholesterol.
