# Supplementary material for: Sleep patterns modify the association of 25(OH)D with poor cardiovascular health in pregnant women
Source: Front Nutr. 2022 Nov 14;9:1013960. doi: 10.3389/fnut.2022.1013960 (PMC9702519; doi:10.3389/fnut.2022.1013960)
Supplement: Supplementary file 1 [file Table_1.DOCX]

Online Supporting Material Page 1

Sleep Patterns Modify the Association of Vitamin D status and Supplementation with Cardiovascular Health in Pregnant Women

Wan-jun Yin, Li-jun Yu, Peng Wang, Rui-xue Tao, Xiao-min Jiang, Ying Zhang, Dao-min Zhu, Peng Zhu

Online Supporting Material Page 2

**Supplemental Figure 1**

9 480 healthy women

212 women without blood sample were excluded

9 886 pregnant women recruited in this prospective birth cohort study

9 674 women with blood sample

9209 women for data analysis in this study

**Supplementary Figure 1** Participants flow chart

264 women were excluded：

34 with severe anemia

28 with heart failure

153 with liver dysfunction

21 with renal dysfunction

28 with thyroid dysfunction

201 women without data of cardiovascular health were excluded

Online Supporting Material Page 3

**Supplemental Figure 2**

|  |  |
| --- | --- |
|  |  |

**Supplemental Figure 2**—The association between serum 25(OH)D concentrations and “clinical” CVH metrics stratified by overall sleep patterns.

HBP, high blood pressure. GDM, gestational diabetes mellitus.

GDM: fasting ≥ 92, 1-h oral glucose tolerance test (OGTT) ≥ 180, 2-h OGTT ≥ 153 mg/dL. HBP: systolic blood pressure ≥ 120 or diastolic blood pressure ≥ 80, high total cholesterol: ≥ 260 mg/dL, overweight: BMI ≥ 28.5 kg/m^2^.

Adjusted for age, education, household income, residence, the season of blood collection, prepregnancy BMI, gestational weight gain rate, parity, depression, family history of diabetes, hypertension, and CVD, smoking, husband's smoking, daily outdoor time, sun exposure, activities, dietary vitamin d intake habits (sea-fish, egg, milk, fungi, red meat, and white meat intake), vitamin D supplementation.

Online Supporting Material Page 4

**Supplemental Table 1—** **Association of sleep behaviors with serum 25(OH)D concentrations and poor CVH**

| Sleep Behaviors | 25(OH)D | |  | Poor CVH | | |
| --- | --- | --- | --- | --- | --- | --- |
|  | M±SD, nmol/L | P-value ^1^ |  | N (%) | P-value ^2^ | RR (95%CI) ^3^ |
| Sleep duration |  | 0.03 |  |  | 0.06 |  |
| Low risk | 40±18 |  |  | 1484 (24.4) |  | 1.00 |
| High risk | 39±19 |  |  | 815 (26.1) |  | 1.12 (1.05, 1.29) |
| Chronotype |  | 0.59 |  |  | 0.07 |  |
| Low risk | 40±18 |  |  | 1464 (24.4) |  | 1.00 |
| High risk | 40±19 |  |  | 835 (26.1) |  | 1.07 (0.96, 1.19) |
| Daytime sleepiness |  | <0.001 |  |  | <0.001 |  |
| Low risk | 41±18 |  |  | 1512 (21.8) |  | 1.00 |
| High risk | 38±18 |  |  | 787 (34.8) |  | 1.91 (1.71, 2.13) |
| Insomnia |  | 0.42 |  |  | 0.001 |  |
| Low risk | 40±18 |  |  | 1706 (24.2) |  | 1.00 |
| High risk | 40±18 |  |  | 593 (27.6) |  | 1.16 (1.03, 1.30) |
| Snoring |  | 0.17 |  |  | <0.001 |  |
| Low risk | 40±18 |  |  | 1606 (23.4) |  | 1.00 |
| High risk | 40±19 |  |  | 693 (29.6) |  | 1.16 (1.04, 1.30) |

^1^ Based on one-way ANOVA; ^2^ Based on chi-square test. ^3^ Based on logistics regression models and adjusted for age, education, household income, residence, the season of blood collection, prepregnancy BMI, gestational weight gain rate, parity, depression, family history of diabetes, hypertension, and CVD, smoking, husband's smoking, daily outdoor time, sun exposure, activities, dietary vitamin d intake habits (sea-fish, egg, milk, fungi, red meat, and white meat intake), vitamin D supplementation.
